# Succession of embryonic and the intestinal bacterial communities of Atlantic salmon (*Salmo salar*) reveals stage‐specific microbial signatures

**DOI:** 10.1002/mbo3.672

**Published:** 2018-06-13

**Authors:** Jep Lokesh, Viswanath Kiron, Detmer Sipkema, Jorge M.O. Fernandes, Truls Moum

**Affiliations:** ^1^ Faculty of Biosciences and Aquaculture Nord University Bodø Norway; ^2^ Laboratory of Microbiology Wageningen University Wageningen The Netherlands

**Keywords:** amplicon sequencing, Atlantic salmon (*Salmo salar*), developmental stages, intestine, microbiome

## Abstract

Host‐associated microbiota undergoes a continuous transition, from the birth to adulthood of the host. These developmental stage‐related transitions could lead to specific microbial signatures that could impact the host biological processes. In this study, the succession of early‐life and intestinal bacterial communities of Atlantic salmon (starting from embryonic stages to 80‐week post hatch; wph) was studied using amplicon sequencing of 16S rRNA. Stage‐specific bacterial community compositions and the progressive transitions of the communities were evident in both the early life and the intestine. The embryonic communities showed lower richness and diversity (Shannon and PD whole tree) compared to the hatchlings. A marked transition of the intestinal communities also occurred during the development; Proteobacteria were dominant in the early stages (both embryonic and intestinal), though the abundant genera under this phylum were stage‐specific. Firmicutes were the most abundant group in the intestine of late freshwater; *Weissella* being the dominant genus at 20 wph and *Anaerofilum* at 62 wph. Proteobacteria regained its dominance after the fish entered seawater. Furthermore, LEfSe analysis identified genera under the above ‐ mentioned phyla that are significant features of specific stages. The environmental (water) bacterial community was significantly different from that of the fish, indicating that the host is a determinant of microbial assemblage. Overall the study demonstrated the community dynamics during the development of Atlantic salmon.

## 1 INTRODUCTION

All animals are born into a microbe‐rich environment, and the host establishes a symbiotic relationship with its microbial community. Such symbiotic relationships play vital roles in the physiological functions of the host (Avella et al., 2012; Mach et al., 2015; Sommer & Bäckhed, 2013; Ye, Amberg, Chapman, Gaikowski, & Liu, 2014). The unstable and compositionally variable microbiota associated with the early life undergoes continuous transitions (from the first few days to the first few years of life) to achieve a compositional profile resembling that of adults (Arrieta, Stiemsma, Amenyogbe, Brown, & Finlay, 2014; Matamoros, Gras‐Leguen, Le Vacon, Potel, & de La Cochetiere, 2013; Rodríguez et al., 2015; Yatsunenko et al., 2012). Microbiota includes bacteria, archaea, fungi, and viruses, and these microorganisms occupy the nutrient‐rich mucosal surfaces of fish (Llewellyn, Boutin, Hoseinifar, & Derome, 2014). Certain groups of microbes that have coevolved with the host to aid in several biological processes are generally considered as the persistent types (Bäckhed et al., 2012; Ursell, Metcalf, Parfrey, & Knight, 2012). On the other hand, the unstable microbial community that colonizes the host under specific biological (e.g., developmental stage) and/or environmental conditions, are the transient groups (Caporaso et al., 2011; Savage, 1977). Although the transition from the early life to the adult microbiome of humans is relatively well documented, the transition during the ontogeny of fish and the establishment of their microbial communities are relatively less explored. Studies on the microbiota of larval Atlantic cod (*Gadus morhua*) and killifish (*Kryptolebias marmoratus*) (Bakke, Coward, Andersen, & Vadstein, 2015; Forberg et al., 2016), and the intestinal microbial communities during the ontogeny of zebrafish (*Danio rerio*) (Stephens et al., 2015; Wong et al., 2015) and catfish (Bledsoe, Peterson, Swanson, & Small, 2016) have shed light on the importance and transformation of early life communities of the fish. Furthermore, the transition of the bacterial composition during the ontogeny of wild Atlantic salmon belonging to different cohorts was described by Llewellyn et al. (2016). High‐throughput sequencing studies have delineated the persistent phyla in Atlantic salmon intestinal bacterial community, which includes members of Firmicutes, Proteobacteria, and Tenericutes (Chiarello, Villéger, Bouvier, Bettarel, & Bouvier, 2015; Dehler, Secombes, & Martin, 2017a; Gajardo et al., 2016; Zarkasi et al., 2014). Furthermore, freshwater to seawater transfer‐related transient (Dehler, Secombes, & Martin, 2017b) and persistent (Rudi et al., 2018) intestinal communities of Atlantic salmon were also described recently.

Atlantic salmon is an anadromous fish of high‐commercial value. In aquaculture production systems, embryos and larvae are maintained in freshwater, and when the fish become smolts (a developmental stage that enables the fish to adapt to its physiological needs in seawater) they are transferred to seawater where they grow into adults. The first feeding starts at ~7–8 weeks post hatching. These events are likely to impact the microbiome of fish and ontogenetic succession of the embryonic and intestinal bacterial communities of Atlantic salmon, until the seawater stage, has not yet been explored in fish originating from the same cohort. The aim of this study was to assess the transition of the bacterial community at the embryonic stages of Atlantic salmon, and in the intestine of the fish from prior to first feeding stage to the 80‐week post hatch stage, employing a 16S rRNA gene‐based phylotyping technique.

## 2 MATERIALS AND METHODS

### 2.1 Biological material

This study was conducted according to the guidelines given by the Norwegian Animal Research Authority (FDU; approval number: 7899). Samples (*n* = 10) from selected life stages of the fish were procured from a local hatchery (Cermaq AS, Hopen, Bodø, Norway). Bacterial communities of the embryonic stages were analyzed from the entire organism. The whole intestine from stages after the yolk sac absorption and the distal intestine from stages thereafter was sampled to understand the changes in the community compositions associated with the ontogeny of the organ. Distal intestinal samples comprising of both mucus and the contents were collected from the late freshwater stages and the seawater stages. All the samples were collected from a single cohort of Atlantic salmon. Stage of the fish, type of sample collected, and the length and weight of the fish at the different sampling time points are summarized in Supporting Information Table S1. The salinity, temperature and pH of the rearing water in the hatchery were <0.5 ppt, 12°C, and 7, respectively. The conditions in the seawater rearing system were 34 ppt, 12–13°C, and 7, respectively.

### 2.2 Sampling

**Figure 1:**
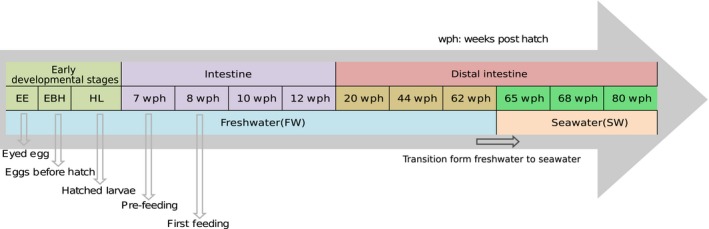
Ontogenetic timeline of salmon depicting the successive developmental stages that were targeted in this study: early developmental stages; early freshwater stages; late freshwater stages; seawater stages. wph: weeks post hatching

Whole organisms (Eyed egg stage ‐ EE, Egg before hatching—EBH and Hatched larvae—HL) were used for the early developmental stages. The embryos were euthanized with 200 mg/L MS‐222 (Tricaine methane sulphonate; Argent Chemical Laboratories, Redmond, USA), transferred to a cryo tube, and then frozen immediately in liquid nitrogen. From 7 weeks post hatch (wph) stage, the fish were dissected, and the whole intestinal samples (7, 8, 10, and 12 wph) were collected aseptically and frozen immediately using liquid nitrogen. At 20 wph, the distal intestine (freshwater stages—20, 44, and 62 wph, seawater stages—65, 68, and 80 wph) was clearly distinguishable and only this intestinal segment was sampled. The distal intestine was cut open longitudinally, and the luminal contents along with the mucus were scraped off using a sterile glass slide and transferred to a cryotube and then frozen in liquid nitrogen. Sampling strategy adopted in the study is illustrated in Figure 1.

Water samples (1L) from the egg incubation trays and the fish holding tanks were also collected and filtered using Supor 200, single 0.2 μm 47 mm filters (Pall Norge AS, Hønefoss, Norway). All the samples and the filters were stored at −80°C before the DNA was extracted.

### 2.3 DNA extraction, preparation of the sequencing libraries (V3–V4 region), library quantification, and sequencing

DNA from the samples was extracted using the QIAamp Fast DNA stool mini kit (Qiagen, Nydalen, Sweden). The frozen samples were thawed in Inhibitex^®^ buffer from the kit and subjected to bead beating using MagNA Lyser (Roche Diagnostics, Oslo, Norway) at 2000 g for 30 s to ensure efficient release of DNA from the microbial communities. The samples were further processed according to the manufacturer's protocol and employing the modifications described earlier by Lokesh & Kiron (2016). DNA from the filters was extracted using the metagenomic DNA isolation kit for water (Epicentre, Middleton, USA) according to the manufacturer's instructions. The DNA samples were quantified using the Qubit^®^ dsDNA BR Assay Kit (Life Technologies, Carlsbad, CA, USA), and their quality was evaluated on an agarose gel.

A paired end, dual index protocol was adopted to amplify and prepare the 16S rRNA gene (V3–V4 regions) sequencing libraries as described by Kozich, Westcott, Baxter, Highlander, & Schloss (2013). The forward primer was modified to contain p5 adapter, i5 index, padF and linkF in addition to the gene specific primer V3‐341F‐ CCTACGGGAGGCAGCAG (Kozich et al., 2013). Similarly, the reverse primer contained p7 adapter, i7 index, pad and linker in addition to the gene specific primer V4‐785R‐ GGACTACHVGGGTWTCTAAT (Kozich et al., 2013). These primers are best suited for targeting the variable regions V3 and V4 with broad phyletic coverage (Klindworth et al., 2013). The DNA samples were normalized to approximately 500 ng/reaction prior to performing PCR. The PCR reactions were performed in a 25 μl reaction volume containing 12.5 μl Kapa HiFi HotStart PCR ReadyMix (KAPA biosystems, Woburn, USA), 2.5 μl each of forward and reverse primers (300 nM), and 7.5 μl of the DNA elutions and water. Thermocycling conditions included initial denaturation at 95**°**C for 5 min, followed by 35 cycles of denaturation at 98**°**C for 30 s, annealing at 58**°**C for 30 s, and extension at 72**°**C for 45 s. A final extension was performed at 72**°**C for 2 min. For each sample, the PCR was run in triplicate. After performing the PCR, the products from the triplicate reactions of a particular sample was pooled and run on a 1.2% agarose gel. The amplified products (~550 bp) were isolated from the gel and purified using the ZR‐96 Zymoclean^™^ Gel DNA Recovery Kit (Irvine, CA, USA) following the manufacturer's instructions. All the primers employed in the study were subjected to a negative PCR (without template) to ascertain the absence of contaminating bacterial DNA either in the primers or the PCR mastermix. The libraries were quantified using the KAPA Library Quantification Kit for Illumina^®^ platforms following the manufacturer's instructions. Libraries were pooled in equimolar (2 nM) concentrations and sequenced using the Illumina MiSeq (San Diego, CA, USA). The final concentration of the library that was used for the sequencing was 9 pM with equimolar 10% PhiX control library.

### 2.4 Data analysis

#### 2.4.1 Quality of the reads

We obtained 7074661 raw sequence reads from the 2 MiSeq runs, split across 127 samples belonging to 13 life stages. FastQ files were used as the input for the UPARSE (usearch version 8.0.1623) pipeline (Edgar, 2013). In both the runs, the percentage of nucleotides with quality score ≥Q30 was very low for the read 4; 37.22 and 43.26 for the sequencing run one and two, respectively (Supporting Information, Table S2). We adopted the maximum expected error filtering strategy as described in the Edgar and Flyvbjerg (Edgar & Flyvbjerg, 2015), to get rid of low quality reads. This quality filtering method performs better than the traditional filtering methods that are based on the Phred (Q) scores. First, with default parameters and maximum expected error set to 1, we merged the forward and the reverse reads. This gave very few sequences per sample due to the large number of erroneous nucleotides in the reverse read. Hence, only the forward reads which contained the V3 region of the 16S rRNA gene were used for further analysis.

#### 2.4.2 Analysis of the microbial community structure

First, the forward reads from different samples were truncated to 200 bp and quality filtered by setting the maximum expected error value to 1. Next, the resulting sequences were dereplicated, abundance sorted and reads with <10 sequences were discarded. OTUs (operational taxonomic units) were clustered at 97% similarity level and then the chimeric sequences were filtered out by UCHIME version 4.2.40 (Edgar, Haas, Clemente, Quince, & Knight, 2011) using the Greengenes reference database gg_13_8 (DeSantis et al., 2006). After the chimera check, the reads (including those with less than 10 sequences) were mapped to the OTUs by searching the reads as query against the OTU representative sequences. Taxonomic ranks were assigned to the OTUs using UTAX algorithm (http://www.drive5.com/usearch/manual/utax_algo.html). UTAX calculates a taxonomy prediction confidence estimate (ranges from 0 to 1) for each of the taxonomic ranks. Assignments with confidence score <0.5 were not considered in the downstream analysis, except for the OTUs that had >10,000 reads, which are indicated using asterisk in the results section. After constructing the OTU table, the OTU count data were split into four categories based on the sample type namely, the early developmental stages, whole intestine from freshwater stages, distal intestine from freshwater stages and distal intestine from seawater stages. All the downstream analyses were performed separately on these four categories. Alpha diversity measures were computed using QIIME (Caporaso et al., 2010) at a rarefaction depth of 2,400 sequences per sample. The significant differences in alpha diversity indices of the groups (one way ANOVA and Tukey's post hoc tests, and unpaired test) were analyzed using GraphPad Prism. Appropriate transformations were employed when the assumptions of normality and equal variances were not met. Beta diversity was assessed based on UniFrac distances (Lozupone & Knight, 2005) and a PCoA plot was created using the R package (R Core Team, 2013) phyloseq version 1.12.2 (McMurdie & Holmes, 2013). Dissimilarities in the community compositions were assessed using ANOSIM−the *p* values and the test statistic R are given in the respective PCoA plots (Ramette, 2007). The significantly abundant OTUs were identified using Linear discriminant analysis effect size (LEfSe) (Segata et al., 2011), which detected the significant (*p* value cut off 0.05 and LDA cut off 3.5) features of the respective groups. The significantly different features were plotted as a cladogram using GraPhlAn (Asnicar, Weingart, Tickle, Huttenhower, & Segata, 2015). The sequence data are deposited in the MG‐rast database, under the project id mgp82105.

## 3 RESULTS

In this study, 4280367 quality filtered sequences were clustered into 1,442 OTUs. The number of OTUs reported in other studies about the intestinal microbiota of Atlantic salmon ranged from 914 to 2,864 (Dehler et al., 2017a, 2017b; Gajardo et al., 2016; Zarkasi et al., 2016). This wide range could be because of the differences in the variable regions sequenced, filtering methods to obtain quality sequences or biological samples (both number and type) or a combination of these factors. Rarefied data, with a depth of 2,400 sequences/sample, were used to calculate the alpha and beta diversity indices. The succession of the ontogeny‐associated microbiota of the four groups is described in this study.

### 3.1 Hatching reflects a shift in the diversity and composition of the microbiota

**Figure 2:**
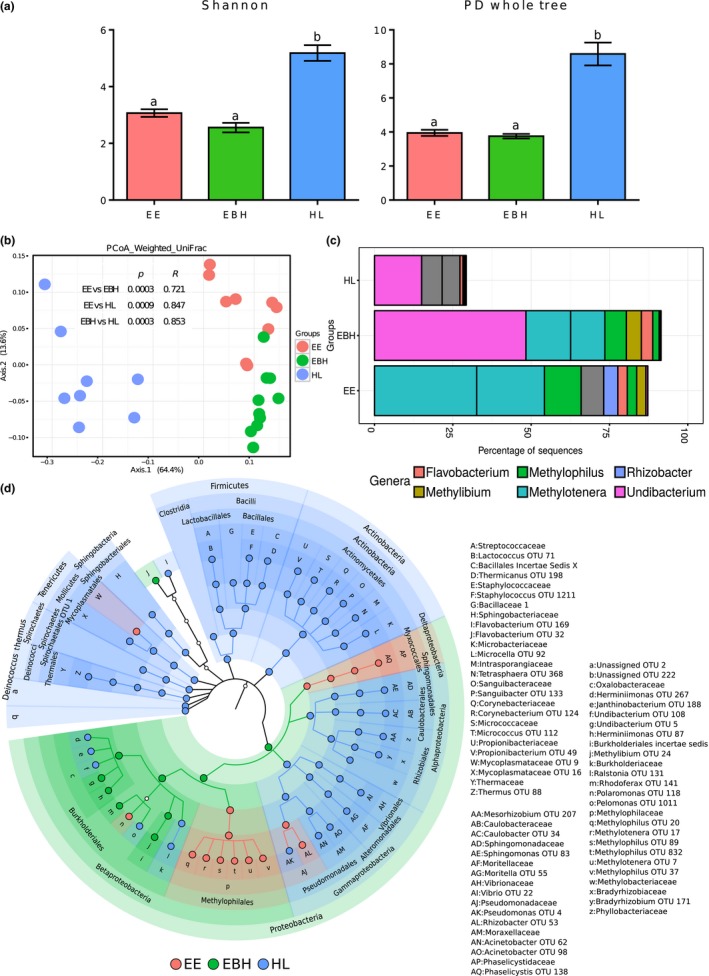
Plots showing the comparisons of microbiota associated with the early developmental stages (EE, EBH, and HL) of Atlantic salmon. Stage‐specific color coding was used for Figures a, b, and d. (a) Alpha diversity indices (Shannon index and PD whole tree) of the bacterial communities and (b) Weighted UniFrac distance‐based PCoA. (c) The mean relative abundance of the 10 most abundant OTUs, plotted at the genus level. The OTUs are colored according to their taxonomic classification, and the OTUs without any assignment are shown in gray. (d) Cladogram showing the significantly abundant taxonomic groups in each of the stages, identified based on the LEfSe analysis (*p* < 0.05 and effect size >3.5).

The diversity indices (Shannon index and PD Whole tree indicating the effective number of common species and phylogenetic diversity) of the communities of HL were significantly higher (*p* < 0.05, Figure 2a) compared with the EE and EBH communities. The community compositions of the early developmental stages were significantly different (Figure 2b; *p* < 0.01, *R* > 0.7, based on weighted UniFrac distances).

*Proteobacteria* (mainly *Methylotenera* and *Undibacterium*) was the abundant phylum in the embryonic stages, and the proportion of the phylum decreased from the EBH to HL stages (Figure 2c). A phylum‐level significant abundance of *Proteobacteria* was evident for the EBH stage due to the abundance of bacteria belonging to all taxonomic levels of Betaproteobacteria. The results obtained for the EE (including *Methylophilales* and *Mycococcales*) and HL stages (*Pseudomonadales*,* Alteromonadales*,* Vibrionales*,* Rhizobiales*,* Caulobacterales,* and *Spingomonadales*) also showed the significantly abundant OTUs under this phylum (Figure 2d). *Methylotenera* and *Methylophilus* were the dominant OTUs in the EE, while *Undibacterium* was the dominant type in EBH and HL. Along with the above‐mentioned bacteria at lower taxonomic levels, *Proteobacteria*,* Actinobacteria*,* Tenericutes*,* Firmicutes*,* Bacteroidetes*,* Deinococcus‐Thermus*,* Spirochaetes* were identified as biomarkers of the HL group (Figure 2d). Most of the OTUs under the order *Burkholderiales* were significantly abundant in either the EBH or the HL (Figure 2d). Furthermore, all genera of *Alpha*‐ and *Gamma‐proteobacteria*, except one OTU belonging to the *Rhizobacter*, were significantly abundant in the HL group (Figure 2d).

### 3.2 Successional changes in the diversity and composition of the intestinal bacterial community of fish at the early freshwater stages

**Figure 3:**
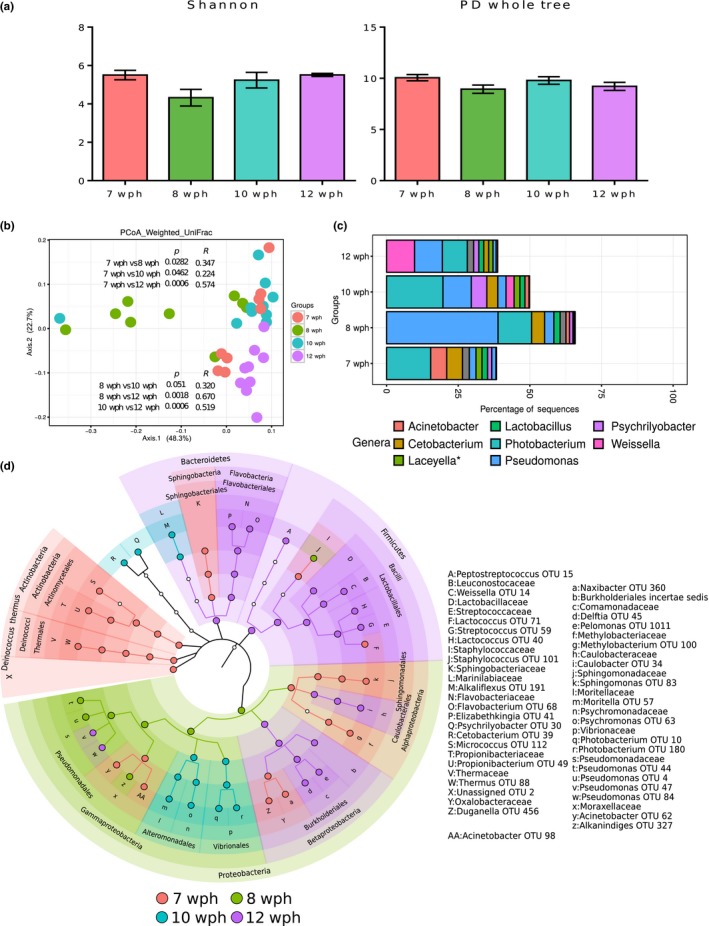
Plots showing the comparisons of the microbiota associated with the whole intestine of Atlantic salmon in freshwater (7, 8, 10, and 12 wph). Stage‐specific color coding is used for Figures a, b, and d. (a) Alpha diversity indices (Shannon index and PD whole tree) of the bacterial communities and (b) Weighted UniFrac distance‐based PCoA. (c) The mean relative abundance of the 10 most abundant OTUs, plotted at the genus level. The OTUs are colored according to their taxonomic classification, and the OTUs without any assignment are shown in gray. (d) Cladogram showing the significantly abundant taxonomic groups in each of the stages, identified based on the LEfSe analysis (*p* < 0.05 and effect size >3.5).

The alpha diversity indices of the communities associated with the intestine of fish at the early freshwater stages did not significantly vary (Figure 3a). The intestinal bacterial communities of the fish at the early freshwater stages were significantly different (Figure 3b; *p* < 0.01, *R* > 0.5; based on weighted UniFrac distances 7, 8, 10 vs. 12 wph).

*Proteobacteria* (including the genera *Photobacterium* (7 wph and 10 wph) and *Pseudomonas* (8 wph)) was the dominant phylum in the first three stages sampled (Figure 3c). However, the genus *Weissella* belonging to the *Firmicutes* was abundant at 12 wph. Aging‐related changes were evident from the significantly abundant OTUs associated with the stages (Figure 3d). The phylum *Proteobacteria* was significantly abundant at 8 wph, primarily reflecting the abundance of the OTUs of the order *Pseudomonadales*, whereas *Vibrionales*,* Alteromonadales,* and the families and genera under these orders were significantly abundant at 10 wph. The significantly abundant OTUs belonging to *Comamonadaceae* under *Burkholderiales* made *Betaproteobacteria* a significant feature at 12 wph, whereas the OTUs of *Oxalobacteriaceae*, belonging to *Betaproteobacteria*, were significantly abundant at 7 wph. *Alphaproteobacteria* was significantly abundant at 7 wph, comprising the OTUs belonging to *Sphingomonadales* and *Methylobacteriaceae*. However, *Caulobacteriales* (*Alphaproteobacteria*) and its members were significantly abundant at 12 wph (Figure 3d). The phyla *Actinobacteria* and *Deinococcus‐Thermus* were significantly abundant at 7 wph (Figure 3d). *Bacteroidetes* were significantly abundant at 12 wph, primarily reflecting the significant abundances of the *Flavobacterial* lineage, whereas the class *Sphingobacteria* (*Bacteroidetes*) was significantly abundant at 7 wph. *Firmicutes* and most of the members of this phylum, particularly the OTUs belonging to the class *Bacilli*, were significantly abundant at 12 wph (Figure 3d).

### 3.3 Successional changes in the diversity and composition of the distal intestinal community of fish at the late freshwater stages

**Figure 4:**
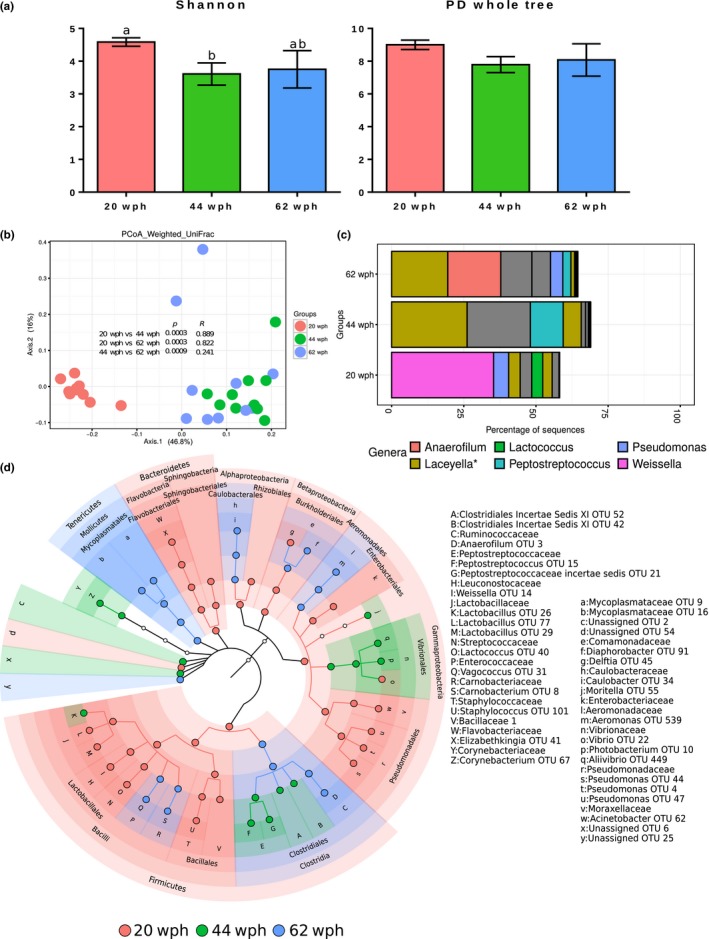
Plots showing the comparison of the microbiota associated with the distal intestine of Atlantic salmon in freshwater (20, 44, and 62 wph). Stage‐specific color coding is used for Figures a, b, and d. (a) Alpha diversity indices (Shannon index and PD whole tree) of the bacterial communities and (b) Weighted UniFrac distance‐based PCoA. (c) The mean relative abundance of the 10 most abundant OTUs, plotted at the genus level. The OTUs are colored according to their taxonomic classification, and the OTUs without any assignment are shown in gray. (d) Cladogram showing the significantly abundant taxonomic groups in each of the stages, identified based on the LEfSe analysis (*p* < 0.05 and effect size >3.5).

The effective number of common species (Shannon index) of the bacterial communities at 44 wph were significantly lower compared to the 20 wph stage (Figure 4a). However, the phylogenetic diversity (PD whole tree) of the communities, did not significantly vary (Figure 4a). The fish at the late freshwater stages had significantly different [Figure 4b; *p* < 0.01, *R* > 0.8, based on weighted UniFrac distances (20 vs. 44, 62 wph)] bacterial communities.

*Firmicutes* (Genera *Weissella* and *Anaerofilum*) was the most dominant phylum in the distal intestine at 20, 44, and 62 wph (Figure 4c). In addition, two OTUs with taxonomy prediction confidence estimates <0.5 (hence excluded from the LEfSe analysis) belonging to the phylum *Firmicutes* (indicated using asterisk, Figure 4c; including the genus *Laceyella,* Figure 4c) were also predominant in this group of fish. The phylum *Firmicutes* and the orders under this group, *Lactobacillales* and *Bacillales*, comprising the class *Bacilli*, were significantly abundant at 20 wph (Figure 4d). The class *Clostridia*, however, was significantly abundant at 62 wph (primarily reflecting one OTU belonging to *Anaerofilum*). Other OTUs belonging to *Peptostreptococcaceae* and some unassigned OTUs under *Clostridiales* were significantly abundant at 44 wph (Figure 4d). While the phylum *Tenericutes* and its members were significantly abundant at 62 wph, the phylum *Bacteroidetes* and its members were significantly abundant at 20 wph. At the phylum level, *Proteobacteria* was not a significant feature of any of the stages. However, the classes under this group (*Alpha*‐, *Beta*‐ and *Gamma‐proteobacteria*) were significant features at 20 wph (Figure 4d). Interestingly, at the order level, the significantly abundant features belonged to different stages, including *Rhizobiales* (20 wph) and *Caulobacteriales* (62 wph) of *Alpha‐proteobacteria*,* Pseudomonadales* (20 wph), *Enterobacteriales* (20 wph), *Vibrionales* (44 wph), and *Aeromonadales* (62 wph) of *Gamma‐proteobacteria* (Figure 4d).

### 3.4 Successional changes in the diversity and composition of the distal intestinal community of seawater fish

**Figure 5:**
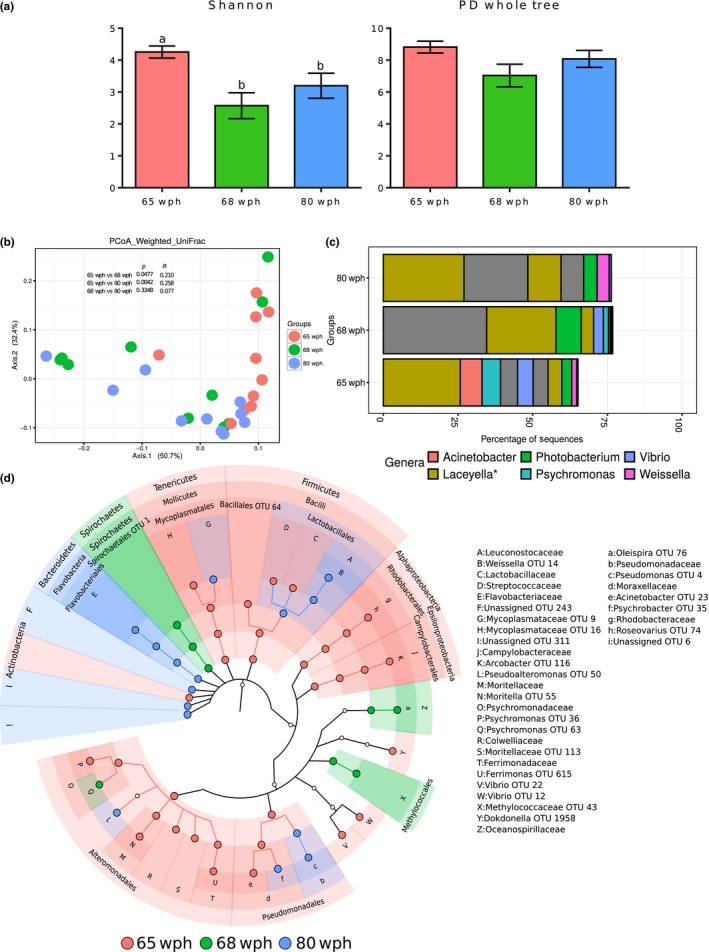
Plots showing the comparison of the microbiota associated with the distal intestine of Atlantic salmon in seawater (65, 68, and 80 wph). Stage‐specific color coding is used for Figures a, b, and d. (a) Alpha diversity indices (Shannon index and PD whole tree) of the bacterial communities and (b) Weighted UniFrac distance‐based PCoA. (c) The mean relative abundance of the 10 most abundant OTUs, plotted at the genus level. The OTUs are colored according to their taxonomic classification, and the OTUs without any assignment are shown in gray. (d) Cladogram showing the significantly abundant taxonomic groups in each of the stages, identified based on the LEfSe analysis (*p* < 0.05 and effect size >3.5).

The effective number of common species (Shannon index) of the communities associated with the distal intestine of the Atlantic salmon in seawater (65, 68, and 80 wph stages) were significantly different (Figure 5a; *p* < 0.05). However, the phylogenetic diversity (PD whole tree) of the three stages was not significantly different. The bacterial community compositions of fish at the seawater stages were not remarkably different as the *R* values were <0.3 (Figure 5b; *p* < 0.01 based on weighted UniFrac distances).

The 2 OTUs (with low‐taxonomic assignment confidence, <0.5) belonging to the genus *Laceyella* (phylum *Firmicutes*) were predominant at 65 and 80 wph (Figure 5c). The phylum *Spirochaetes* (with unassigned taxonomy at genus level) was predominant in the distal intestine at 68 wph. *Actinobacteria*,* Tenericutes,* and *Firmicutes* were the significantly abundant phyla at 65 wph. *Spirochaetes* and *Bacteroidetes* were the significant phyla at 68 and 80 wph, respectively (Figure 5d). Under *Firmicutes*, one OTU belonging to *Weissella* was a feature of the 80 wph, making *Lactobacillales* a significant feature at 80 wph. Although at 65 wph more significantly abundant taxonomic biomarkers were observed for the phylum *Proteobacteria*, phylum‐level significant abundance was not detected. The classes *Alpha‐proteobacteria*,* Epsilon‐proteobacteria* and their members were significantly abundant at 65 wph (Figure 5d). Under *Proteobacteria*, the orders *Alteromonadales*,* Pseudomonadales* and 2 OTUs belonging to the genus *Vibrio* were significantly abundant at 65 wph (Figure 5d). Under *Pseudomonadales*, two OTUs of *Psychrobacter* and *Pseudomonas* were the significantly abundant features at 80 wph (Figure 5d). The bacterial compositional shift at the phylum‐level is shown in Supporting Information Figure S1.

### 3.5 Transition in the communities associated with the distal intestine during transfer from freshwater to seawater

**Figure 6:**
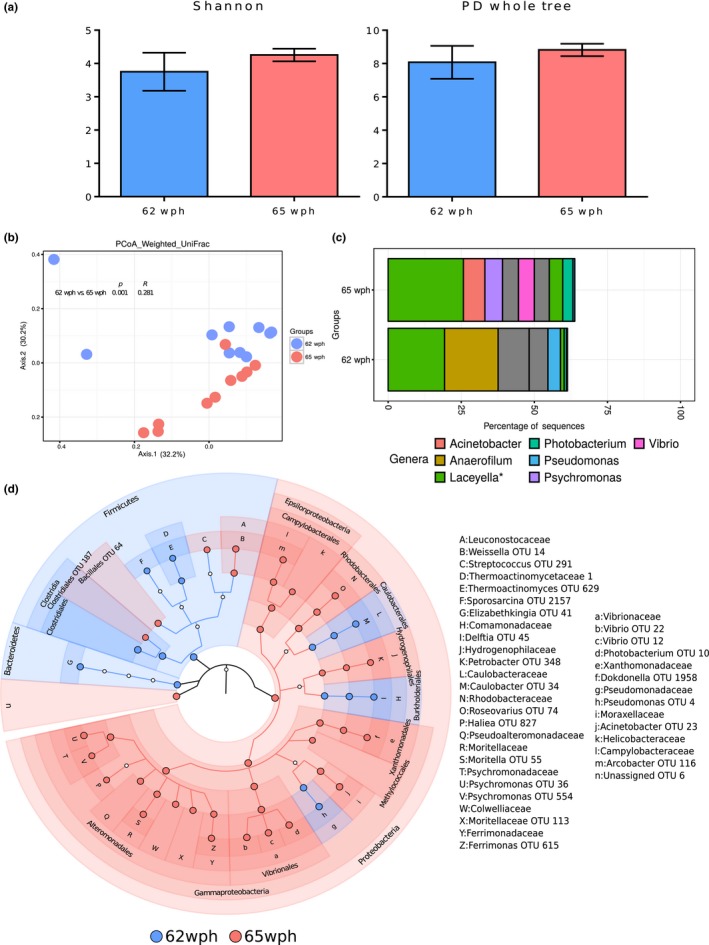
Plots showing the comparison between stages 62wph—freshwater and 65wph—seawater. A stage‐specific color coding is used for figures a, b, and d. (a) Alpha diversity indices (Shannon index, PD whole tree) of the bacterial communities and (b) Weighted UniFrac distances‐based PCoA. (c) Mean relative abundance of the 10 most abundant OTUs, plotted at the genus level. The OTUs are colored according to their taxonomic classification, and the OTUs without any assignment are shown in gray. (d) Cladogram showing the significantly abundant taxonomic groups in each of the stages, identified based on the LEfSe analysis (*p* < 0.05 and effect size >3.5).

There were no significant differences in the diversity indices based on the Shannon index and PD whole tree values (Figure 6a; *p* > 0.05) associated with 62 and 65 wph. Beta diversity of the bacterial communities was significantly different between the groups (*p* < 0.01, but the R value was low (*R* < 0.3).

*Firmicutes* (Laceyella* and *Anaerofilum*), *Tenericutes* (Unassigned genera), and *Proteobacteria* (*Photobacterium*) were the dominant phyla at the two stages examined (Figure 6c). *Bacteroidetes* and *Firmicutes* were abundant at 62 wph (freshwater), whereas *Proteobacteria* was significantly abundant at 65 wph (seawater) (Figure 6d). Some members of *Proteobacteria* namely, *Caulobacterales*,* Burkholderiales* and *Pseudomonadaceae* were the abundant features at 62 wph (Figure 6d). The OTUs under *Firmicutes*, including *Clostridiales*,* Bacillales*,* Streptococcus* and *Leuconostocaceae*, were the significant features at 65 wph.

The bacterial communities of the water samples and the corresponding fish‐associated microbiota were different (Supporting Information, Figure S2).

## 4 DISCUSSION

This study profiled the progressive transition of the bacterial communities of Atlantic salmon: in the early embryonic stages (EE, EBH, and HL), in the entire intestine of the early freshwater stages (7, 8, 10, 12 wph), and in the distal intestine of the late freshwater stages (20, 44, 62 wph), and in the distal intestine of the seawater stages (65, 68 and 80 wph).

Fish eggs are colonized by diverse microbial communities (Hansen, 1999; Llewellyn et al., 2014). In this study, the bacterial community associated with the whole organism was examined up to the hatching stage. The transition from eyed eggs (EE) to those prior to hatching (EBH) was characterized based on changes in both richness and phylogenetic diversity as well as the composition, particularly at the genus level: *Methylotenera* and *Methylophilus* were dominant in the EE, whereas *Undibacterium* was dominant in the EBH and HL groups. Previous reports have not clearly described the presence and biological functions of these genera on fish eggs though *Methylotenera* has been found abundant on the brown trout (*Salmo trutta*) embryos (Wilkins, Fumagalli, & Wedekind, 2016). The abovementioned communities of the phylum Proteobacteria are likely to be egg surface‐specific (Fujimoto, Crossman, Scribner, & Marsh, 2013; Llewellyn et al., 2014; Yoshimizu, Kimura, & Sakai, 1980) and the mechanisms causing the shift in the genera are not clear yet, although neutral and nonneutral assembly models have been proposed for zebrafish (Burns et al., 2016). As zebrafish ages, the assembly of the associated bacterial community is not decided according to chance and dispersal, but through microbial interactions, active dispersal, or host selection (Burns et al., 2016). The hatchling‐associated community was significantly diverse (phylogenetically) compared with the communities prior to hatching. Hatching is a critical process because the sterile embryo contacts the microbe‐rich environment (Fujimoto et al., 2013; Galindo‐Villegas, Garcia‐Moreno, de Oliveira, Meseguer, & Mulero, 2012; Zapata, Diez, Cejalvo, Gutiérrez‐de Frías, & Cortés, 2006) when the immune system of the organism is still immature in terms of its ability to mount an adaptive response (Zapata et al., 2006). The diverse community members associated with hatchlings might aid the host in defence against pathogens (Liu et al., 2014; Llewellyn et al., 2014; Rawls, Samuel, & Gordon, 2004). From the hatching stage onward, major mucosal organs, such as the gills, skin and gut physically come in contact with the environmental microbes providing specific niche for phylotypes that can colonize these tissues because it is previously shown that the microbial profiles in these tissues are distinct (Lowrey, Woodhams, Tacchi, & Salinas, 2015). The specific phylotypes that colonize these microenvironments might play key roles in the normal development of these organs (Bates et al., 2006; Chung et al., 2012; Ingerslev et al., 2014; Llewellyn et al., 2014; Rawls et al., 2004). In addition, at this stage, oxygen uptake changes from cutaneous to pharyngeal (Wells & Pinder, 1996), and this development could affect the community composition. These ontogenic changes might contribute to the HL‐associated diverse bacterial community, which had significantly abundant bacteria belonging to *Proteobacteria*,* Actinobacteria*,* Tenericutes*,* Firmicutes*,* Bacteroidetes*,* Deinococcus‐Thermus,* and *Spirochaetes*. Moreover, the early life communities could be species‐ and stage‐specific as shown previously in Atlantic cod and halibut eggs. *Vibrio fischeri* and *Leucothrix mucor* were abundant on cod eggs, whereas *Moraxella* and *Alcaligens* were abundant on halibut eggs (Hansen & Olafsen, 1989). In addition, microbiota of cod larvae was highly distinct from those of their environment and live feed indicating the selection by the host (Bakke et al., 2013; Stephens et al., 2015; Yan et al., 2016). Atlantic salmon embryonic stages had significantly abundant *Methylotenera*,* Methylophilus* and *Undibacterium*. All these abundant bacteria associated with the fish eggs belong to the phylum *Proteobacteria*.

Yan et al. (2016), have introduced the “fish gut island” ecosystem theory and hypothesized that host factors (Li, Yu, Feng, Yan, & Gong, 2012; Li et al., 2014; Rawls, Mahowald, Ley, & Gordon, 2006; Roeselers et al., 2011; Yan et al., 2016), rather than the environment, are the major deterministic filters that decide the microbial assemblage in the fish intestine. In this study, we analyzed the bacterial communities in the water from which the corresponding developmental stages were collected. It was previously reported that the gut microbiota of the aquacultured species (grass carp, *Ctenopharyngodon idella*; Chinese perch, *Siniperca chuatsi*; and southern catfish, *Silurus meridionalis*) from the same regional pool are similar, and these fishes have developmental stage‐dependent communities, which are distinct from those of the rearing water (Li et al., 2012). In this study, the water and fish‐associated bacterial community composition and abundance were not identical, as described by Yan et al. (2016), and the authors indicate that the host is the principal factor that account for the modulation of the microbiota. Other investigations (Li et al., 2012, 2014; Rawls et al., 2006; Roeselers et al., 2011) on fish gut microbiota also suggested that the host considerably affects the community composition and turnover patterns. Hence, it is plausible that deterministic processes can regulate the succession of the bacterial communities of Atlantic salmon.

After the formation of the gut, that is, 7 weeks after hatching, the bacterial community associated with the whole intestine was assessed. Neither the effective number of common species nor the bacterial lineages associated with these stages were significantly different. Feeding led to a transition of the rainbow trout larval intestine from a *Bacteroidetes*‐dominant to a *Firmicutes*‐ and *Proteobacteria*‐dominant community (Ingerslev et al., 2014). The observations in this study suggest that feeding causes a phylum‐level shift to *Proteobacteria* (at 8 wph) and *Bacteroidetes* (as a result of the *Flavobacterial* lineage, at 12wph), and *Firmicutes* (primarily reflecting the abundance of the genus *Weissella*, at 12 wph). Age‐related transitions (from parr to adult stage) in the intestinal communities of the wild Atlantic salmon belonging to different cohorts have been previously hypothesized to be controlled by both deterministic and stochastic factors (Llewellyn et al., 2016) because life cycle stages rather than the geography had large impact on the composition. A similar finding was reported in a freshwater carnivore, channel catfish (Bledsoe et al., 2016); a transition in the community composition was observed across developmental stages.

The distal intestine was clearly distinguishable at 20 wph; therefore, the bacterial community associated with this intestinal region was analyzed from this time point. The effective number of common species of the two stages 20 and 44 wph were significantly different, reflecting the lower richness of the community at 44 wph. However, the lineages of the bacterial communities were not far apart as there was no difference in the phylogenetic diversity of the communities. In this study, the phylum *Firmicutes* was significantly abundant at 12 wph, during the ontogeny of the intestine and after differentiation of the distal intestine, that is, at 20 wph. The genera *Weissella*, Laceyella*, and *Anaerofilum* were the predominant contributors to the significant abundance of *Firmicutes*. Rainbow trout, also presents a high abundance of *Firmicutes*, with OTUs belonging to Bacilli as the predominant type (Wong et al., 2013), similar to the fish at 20 wph. In contrast, members of *Bacilli* were not abundant in the gut of the cyprinids common carp (*Cyprinus carpio*) and zebrafish (*Danio rerio*) (van Kessel et al., 2011; Roeselers et al., 2011). Bacteria belonging to *Clostridia* was significantly abundant once the fish was ready to enter the seawater, that is, at 62 wph. These findings may be indicating the importance of *Firmicutes* to trout and salmon. The phylum *Tenericutes* also became significantly abundant just prior to the transfer of the fish to seawater (at 62 wph) and it remained as the significant feature even after entry into seawater (at 65 wph), as observed in other studies with Atlantic salmon (Holben et al., 2002; Llewellyn et al., 2016) and trout (Lowrey et al., 2015). Higher abundance of intestinal Tenericutes (especially *Mycoplasma*) appears to be a characteristic of both farmed fish belonging to the same cohort and wild marine adults belonging to multiple cohorts (Holben et al., 2002; Llewellyn et al., 2016).

*Firmicutes* were significantly abundant soon after the fish were transferred to seawater, and the OTUs belonging to Laceyella* remained predominant. In addition, the OTUs belonging to *Spirochaetes*,* Proteobacteria* and *Tenericutes* were also prominent. The significant abundance of the phylum *Spirochaetes* at 68 wph suggests their importance for carnivorous fish. Similar to the findings in this study, *Spirochaetes* were highly abundant in other carnivorous fish, including mahi‐mahi (*Coryphaena hippurus*) and great barracuda (*Sphyraena barracuda*) (Givens, Ransom, Bano, & Hollibaugh, 2015). During the seawater stages, the effective number of common species in the distal intestinal community significantly decreased with time. Similar diversity shift and the overabundance of the few phylotypes in the microbiota of the intestine (Llewellyn et al., 2016) and skin (Lokesh & Kiron, 2016) of adult Atlantic salmon and rainbow trout gut (Lowrey et al., 2015), respectively, have been previously documented. Changes in the phylum *Tenericutes* (mainly *Mycoplasma* spp.) during development were minimal in this study. Although *Tenericutes* were part of the microbiota at the early developmental stages and were significantly abundant in the HL group and the distal intestine at 62 and 65 wph, the proportion of this phylum (20% at 62 wph) was much less compared with the study by Holben et al. (2002), who reported 70%–90% *Tenericutes* in most of their samples that originated from both farmed and wild fish. Another study on the transition in the community composition of the wild Atlantic salmon by Llewellyn et al. (2016) showed that the proportion of *Mycoplasma* spp. increased consistently with development and it was most abundant in the adults returning for spawning. On the other hand, previous reports on the abundance of *Mycoplasma* spp. in the intestine are contrasting; Llewellyn et al., (2016) and Holben et al. (2002) found an over dominance, whereas Zarkasi et al. (2014, 2016) detected only sporadic occurrence of the species. These discrepancies could be because of the genetic background or the geographical locations of the fish sampled. In addition, it has been previously shown that the microbial richness in the intestine of wild Atlantic salmon belonging to multiple cohorts decreased as the fish became older (freshwater returning adults) (Llewellyn et al., 2016). The individuals in this study belonged to a single cohort and we observed a decrease in the richness after the fish were transferred to seawater (68 wph and 80 wph). It is unlikely that this decrease in richness is linked to starvation as hypothesized by Llewellyn et al. (2016) because the fish in this study were fed during the experimental period.

We also examined the diversity and differences in the significantly abundant phyla associated with the distal intestine of the fish in freshwater and seawater by comparing 62 wph versus 65 wph. Though this comparison did not reveal significant differences in the richness, phylogenetic diversity and beta diversity of the communities, LEfSe analyses revealed significantly different abundances (even at phylum‐level) associated with a particular group. *Proteobacteria,* which were abundant in the early stages regained their dominance when the fish were introduced into seawater, whereas *Firmicutes* and the *Bacteroidetes* were significant features of the freshwater group. This transition to a *Proteobacteria*‐rich community when the fish enters seawater has been previously recorded; in both fish skin (Lokesh & Kiron, 2016; Schmidt, Smith, Melvin, & Amaral‐Zettler, 2015) and intestinal (both mucosa and digesta) microbiota (Gajardo et al., 2016). A meta‐analysis also revealed the differences in the gut bacterial community compositions of freshwater and the seawater fishes (Sullam et al., 2012). Dehler et al. (2017b) demonstrated a similar trend in the transition of the community; the abundance of Proteobacteria increased when the fish belonging to the same cohort entered seawater. On the contrary, Rudi et al. (2018), reported that the freshwater to seawater transition results in an increased abundance of intestinal Firmicutes and a decrease in Proteobacteria and Actinobacteria. The transition‐linked abundance of specific groups are quite relevant for the aquaculture industry because during this critical phase the fish undergoes several physiological changes (McCormick, Hansen, Quinn, & Saunders, 1998). The changes in the community profile observed in these studies should be further explored to understand if such alterations make the fish susceptible to diseases.

## 5 CONCLUSION

This study examined the transition of the embryonic and intestinal bacterial communities of Atlantic salmon. Stage‐specific microbial signatures were evident at the phylum level. *Proteobacteria* was the most abundant phylum in eggs. The diversity of the hatchling‐associated community increased, reflecting the significant abundance of *Actinobacteria*,* Firmicutes*,* Tenericutes*,* Spirochaetes,* and *Deinococcus‐Thermus*. In the intestine of the fish at the early freshwater stages, *Proteobacteria* was dominant. Subsequently *Firmicutes* and *Bacteroidetes* became the significantly abundant phyla. Although the former group dominated in the distal intestine of the fish at the late freshwater stages, *Proteobacteria* again became the significantly abundant phylum after the fish were in seawater. Specific phylum can be employed as indicators of the developmental stages of Atlantic salmon. After confirming the functional significance of these indicators, selected members of a particular phylum can be enriched through microbial manipulation for better growth and health of farmed fish. Overall, these results provide basic knowledge required for the development of sustainable health ‐ promoting microbial manipulation strategies for the salmon farming industry.

## CONFLICT OF INTEREST

The authors have no conflict of interest to declare.
