# Supplementary material for: Succession of embryonic and the intestinal bacterial communities of Atlantic salmon (Salmo salar) reveals stage‐specific microbial signatures
Source: Microbiologyopen. 2018 Jun 13;8(4):e00672. doi: 10.1002/mbo3.672 (PMC6460355; doi:10.1002/mbo3.672)
Supplement: Supplementary file 1 [file MBO3-8-e00672-s001.docx]

**Table S1**: Sample metadata including the description of the sample IDs used, length and weight of the fish sampled at different stages.

| Group_ID | Stage of development | Type of sample | Environment | Average length in cm (mean±SE) | Average weight in gram (mean±SE) |
| --- | --- | --- | --- | --- | --- |
| EE | Eyed egg (Primordial  intestine) | Whole animal | Freshwater | NA | 0.1534 ± 0.004 |
| EBH | Pre hatching | Whole animal | Freshwater | NA | 0.1575 ± 0.003 |
| HL | Post hatching | Whole animal | Freshwater | 1 ± 0.02 | 0.1611 ± 0.005 |
| 7wph | 7 Weeks post hatching | Intestine | Freshwater | 2.77 ± 0.04 | 0.1985 ± 0.009 |
| 8wph | 8 Weeks post hatching | Intestine | Freshwater | 2.92 ± 0.02 | 0.2388 ± 0.013 |
| 10wph | 10 Weeks post hatching | Intestine | Freshwater | 3.64 ± 0.07 | 0.5657 ± 0.027 |
| 12wph | 12 Weeks post hatching | Intestine | Freshwater | 4.87 ± 0.07 | 1.173 ± 0.059 |
| 20wph | 20 Weeks post hatching | Distal intestine | Freshwater | 11 ± 0.21 | 14.7 ± 0.746 |
| 44wph | 44 Weeks post hatching | Distal intestine | Freshwater | 18.85 ± 0.63 | 63.1 ± 0.948 |
| 62wph | 62 Weeks post hatching | Distal intestine | Freshwater | 19.25 ± 0.34 | 70.71 ± 2.603 |
| 65wph | 65 Weeks post hatching | Distal intestine | Sea water | 21.45 ± 0.40 | 84.1 ± 5.386 |
| 68wph | 68 Weeks post hatching | Distal intestine | Sea water | 22.1 ± 0.51 | 87.4 ± 5.209 |
| 80wph | 80 Weeks post hatching | Distal intestine | Sea water | 22.49 ± 0.31 | 97.9 ± 3.223 |

**Table S2**: Read statistics corresponding to the 2 MiSeq sequencing runs.

| **Run_1_Summary** | | | | | | | | |
| --- | --- | --- | --- | --- | --- | --- | --- | --- |
|  |  | **Yield Total** | **Projected Total Yield** | **Aligned** | **Error Rate** | **Intensity** | **% >=** | **Number of reads** |
| **Level** | **Cycles** | **(G)** | **(G)** | **(%)** | **(%)** | **Cycle 1** | **Q30** | **(M)** |
| Read 1 | 301 | 3.07 | 3.07 | 10.27 | 3.72 | 44 | 71.81 | 11.41 |
| Read 2 (I) | 8 | 0.07 | 0.07 | 0 | 0 | 224 | 80.92 |  |
| Read 3 (I) | 8 | 0.07 | 0.07 | 0 | 0 | 298 | 89.27 |  |
| Read 4  Non-Indexed Total | 301  602 | 3.07  6.14 | 3.07  6.14 | 9.51  9.89 | 6.32  5.02 | 52  48 | 37.22  54.51 |  |
| Total | 618 | 6.29 | 6.29 | 9.89 | 5.02 | 155 | 55.21 |  |
| **Run_2_Summary** | | | | | | | | |
|  |  | **Yield Total** | **Projected Total Yield** | **Aligned** | **Error Rate** | **Intensity** | **% >=** | **Number of reads** |
| **Level** | **Cycles** | **(G)** | **(G)** | **(%)** | **(%)** | **Cycle 1** | **Q30** | **(M)** |
| Read 1 | 301 | 3.62 | 3.62 | 10.63 | 3.1 | 49 | 75.35 | 15.99 |
| Read 2 (I) | 8 | 0.08 | 0.08 | 0 | 0 | 211 | 79.34 |  |
| Read 3 (I) | 8 | 0.08 | 0.08 | 0 | 0 | 316 | 88.4 |  |
| Read 4  Non-Indexed Total | 301  602 | 3.62  7.24 | 3.62  7.24 | 10.31  10.47 | 4.15  3.63 | 52  50 | 43.26  59.31 |  |
| Total | 618 | 7.41 | 7.41 | 10.47 | 3.63 | 157 | 59.87 |  |

**Read statistics after quality filter**

Number of samples: 128 Counts/sample summary:

Number of observations: 1442 Min: 925.0

Total count: 4280367 Max: 218888.0

Median: 25458.000

Mean: 33440.367

Std. Dev.: 31143.326
