# Supplementary material for: Succession of embryonic and the intestinal bacterial communities of Atlantic salmon (Salmo salar) reveals stage‐specific microbial signatures
Source: Microbiologyopen. 2018 Jun 13;8(4):e00672. doi: 10.1002/mbo3.672 (PMC6460355; doi:10.1002/mbo3.672)
Supplement: Supplementary file 2 [file MBO3-8-e00672-s002.pdf]

Supplementary materials

Succession of embryonic and the intestinal bacterial communities of Atlantic salmon (*Salmo salar*) reveals stage-specific microbial signatures

Jep Lokesh[a], Viswanath Kiron[a,#], Detmer Sipkema[b], Jorge M.O. Fernandes[a], Truls Moum[a]

Faculty of Biosciences and Aquaculture, Nord University, Bodø, Norway[a]; Laboratory of Microbiology, Wageningen University, Wageningen, The Netherlands[b]

Running title: Embryonic and intestinal microbiota of Atlantic salmon

#Address correspondence to Viswanath Kiron, Nord University, Universitetsalléen 11, 8049 Bodø, Norway. Tel. +47 75517399; Email: kiron.viswanath@nord.no

Keywords: Aquaculture, Atlantic salmon (*Salmo salar*), developmental stages, microbiome, intestine, amplicon sequencing

Supplementary figures

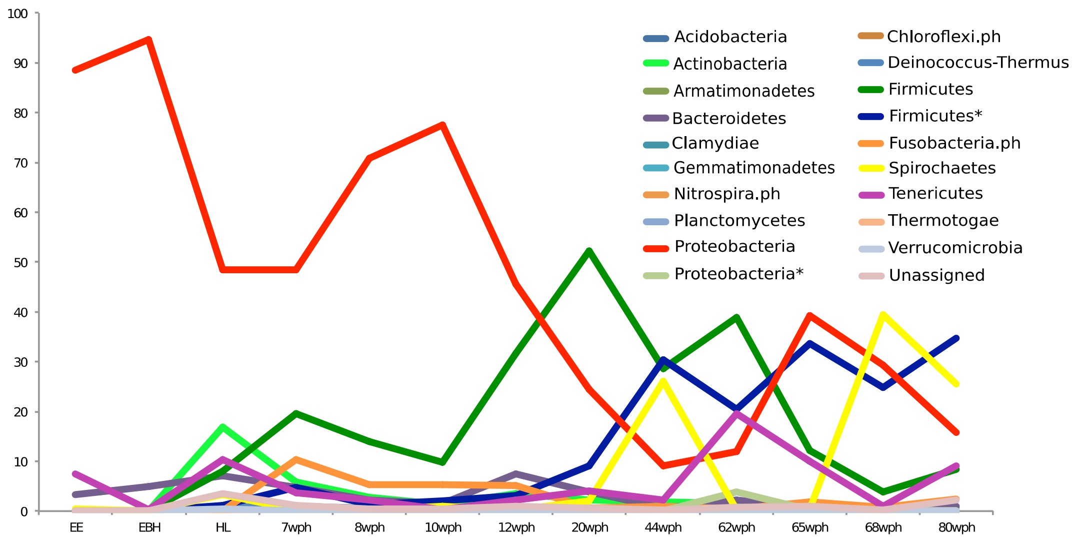

Fig S1. Overview of the phylum-level shifts in the bacterial communities at the different life stages of Atlantic salmon originating from a single cohort. * indicates that phyla with taxonomy assignment confidence below 0.5.

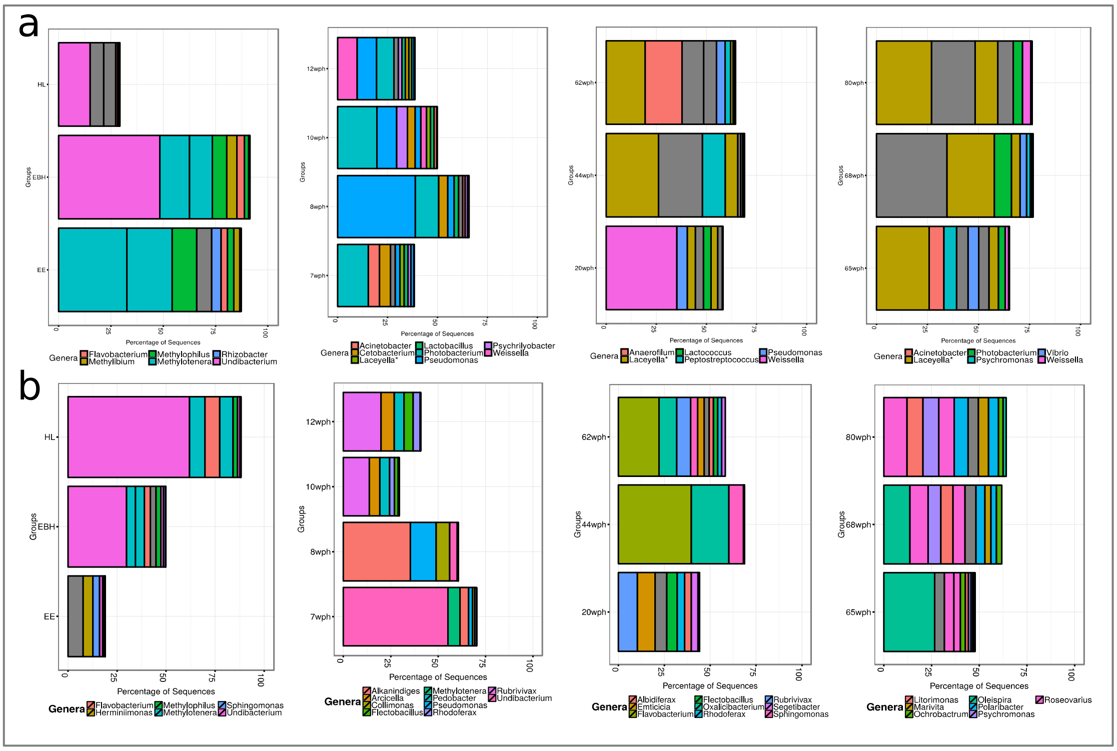

Fig S2. Composition of the bacterial communities of the fish (a) and water (b) collected at respective stages of development. Ten most abundant OTUs from each sampling point are plotted. It is evident that the most abundant communities of the fish and water are different.

Table S1: Sample metadata including the description of the sample IDs used, length and weight of the fish sampled at different stages.

Group_ID	Stage of development	Type of sample	Environment	Average length in cm (mean±SE)	Average weight in gram (mean±SE)
EE	Eyed egg (Primordial intestine)	Whole animal	Freshwater	NA	0.1534 ± 0.004
EBH	Pre hatching	Whole animal	Freshwater	NA	0.1575 ± 0.003
HL	Post hatching	Whole animal	Freshwater	1 ± 0.02	0.1611 ± 0.005
7wph	7 Weeks post hatching	Intestine	Freshwater	2.77 ± 0.04	0.1985 ± 0.009
8wph	8 Weeks post hatching	Intestine	Freshwater	2.92 ± 0.02	0.2388 ± 0.013
10wph	10 Weeks post hatching	Intestine	Freshwater	3.64 ± 0.07	0.5657 ± 0.027
12wph	12 Weeks post hatching	Intestine	Freshwater	4.87 ± 0.07	1.173 ± 0.059
20wph	20 Weeks post hatching	Distal intestine	Freshwater	11 ± 0.21	14.7 ± 0.746
44wph	44 Weeks post hatching	Distal intestine	Freshwater	18.85 ± 0.63	63.1 ± 0.948
62wph	62 Weeks post hatching	Distal intestine	Freshwater	19.25 ± 0.34	70.71 ± 2.603
65wph	65 Weeks post hatching	Distal intestine	Sea water	21.45 ± 0.40	84.1 ± 5.386
68wph	68 Weeks post hatching	Distal intestine	Sea water	22.1 ± 0.51	87.4 ± 5.209
80wph	80 Weeks post hatching	Distal intestine	Sea water	22.49 ± 0.31	97.9 ± 3.223

Table S2: Read statistics corresponding to the 2 MiSeq sequencing runs.

Run_1_Summary

Level	Cycles	Yield Total (G)	Projected Total Yield (G)	Aligned (%)	Error Rate (%)	Intensity Cycle 1	% >= Q30	Number of reads (M)
Read 1	301	3.07	3.07	10.27	3.72	44	71.81	11.41
Read 2 (I)	8	0.07	0.07	0	0	224	80.92	
Read 3 (I)	8	0.07	0.07	0	0	298	89.27	
Read 4	301	3.07	3.07	9.51	6.32	52	37.22	
Non-Indexed Total	602	6.14	6.14	9.89	5.02	48	54.51	
Total	618	6.29	6.29	9.89	5.02	155	55.21	

Run_2_Summary

Level	Cycles	Yield Total (G)	Projected Total Yield (G)	Aligned (%)	Error Rate (%)	Intensity Cycle 1	% >= Q30	Number of reads (M)
Read 1	301	3.62	3.62	10.63	3.1	49	75.35	15.99
Read 2 (I)	8	0.08	0.08	0	0	211	79.34	
Read 3 (I)	8	0.08	0.08	0	0	316	88.4	
Read 4	301	3.62	3.62	10.31	4.15	52	43.26	
Non-Indexed Total	602	7.24	7.24	10.47	3.63	50	59.31	
Total	618	7.41	7.41	10.47	3.63	157	59.87	

Read statistics after quality filtering

Num samples: 128	Counts/sample summary:
Num observations: 1442	Min: 925.0
Total count: 4280367	Max: 218888.0
	Median: 25458.000
	Mean: 33440.367
	Std. Dev.: 31143.326
